# Supplementary material for: Host starvation and in hospite degradation of algal symbionts shape the heat stress response of the Cassiopea-Symbiodiniaceae symbiosis
Source: Microbiome. 2024 Feb 29;12:42. doi: 10.1186/s40168-023-01738-0 (PMC10902967; doi:10.1186/s40168-023-01738-0)
Supplement: Supplementary file 2 — Additional file 1: Figure S1. Schematic presentation of the experimental setup viewed from the side and from above with sampling design information. Figure S2. Photos of the medusae collected at SP2 on day 1 and 10 of the experiment in the control treatment, and heat stress treatment. Figure S3. Impact of heat stress on chlorophyll a content and algal symbiont density per wet weight. Figure S4. Temperature effects on the enrichment from assimilation of isotopically labeled bicarbonate and ammonium in the epidermis of heat-stressed medusae. Figure S5. SEM and 12C2- and 14N12C- NanoSIMS images corresponding to the correlative SEM and NanoSIMS images presented in Fig. 3. Figure S6. Tissue and cellular ultrastructure of control and heat-stressed medusae bells at SP2 imaged by light microscopy with H&E staining and SEM. Table S1. Number of NanoSIMS images per biological replicate and the associated number of regions of interest (ROIs) per Cassiopea compartment defined for the isotopic enrichment analyses. [file 40168_2023_1738_MOESM1_ESM.pdf]

Supplementary data

Host starvation and *in hospite* degradation of algal symbionts shape the heat stress response of the *Cassiopea*-Symbiodiniaceae symbiosis

Gaëlle Toullec[1], Nils Rädecker[1], Claudia Pogoreutz[1,2], Guilhem Banc-Prandi[1], Stéphane Escrig[1], Christel Genoud[3], Cristina Martin Olmos[1,4], Jorge Spangenberg[5], Anders Meibom[1,4].

This document includes the supplementary data Figures S1, S2, S3, S4, S5, S6 and Table S1.

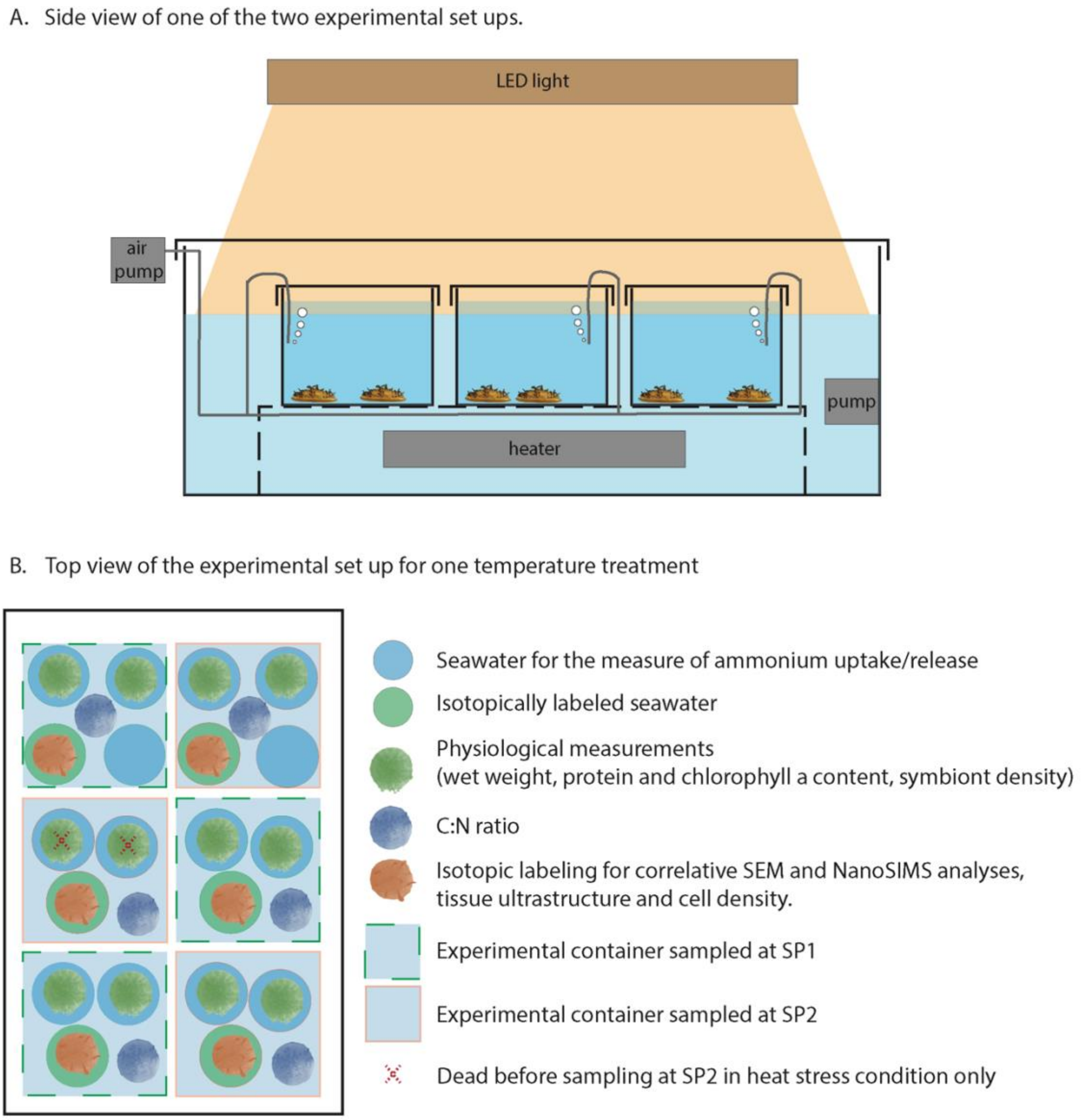

Figure S1: Schematic presentation of the experimental setup viewed from the side (A) and from above with sampling design information (B).

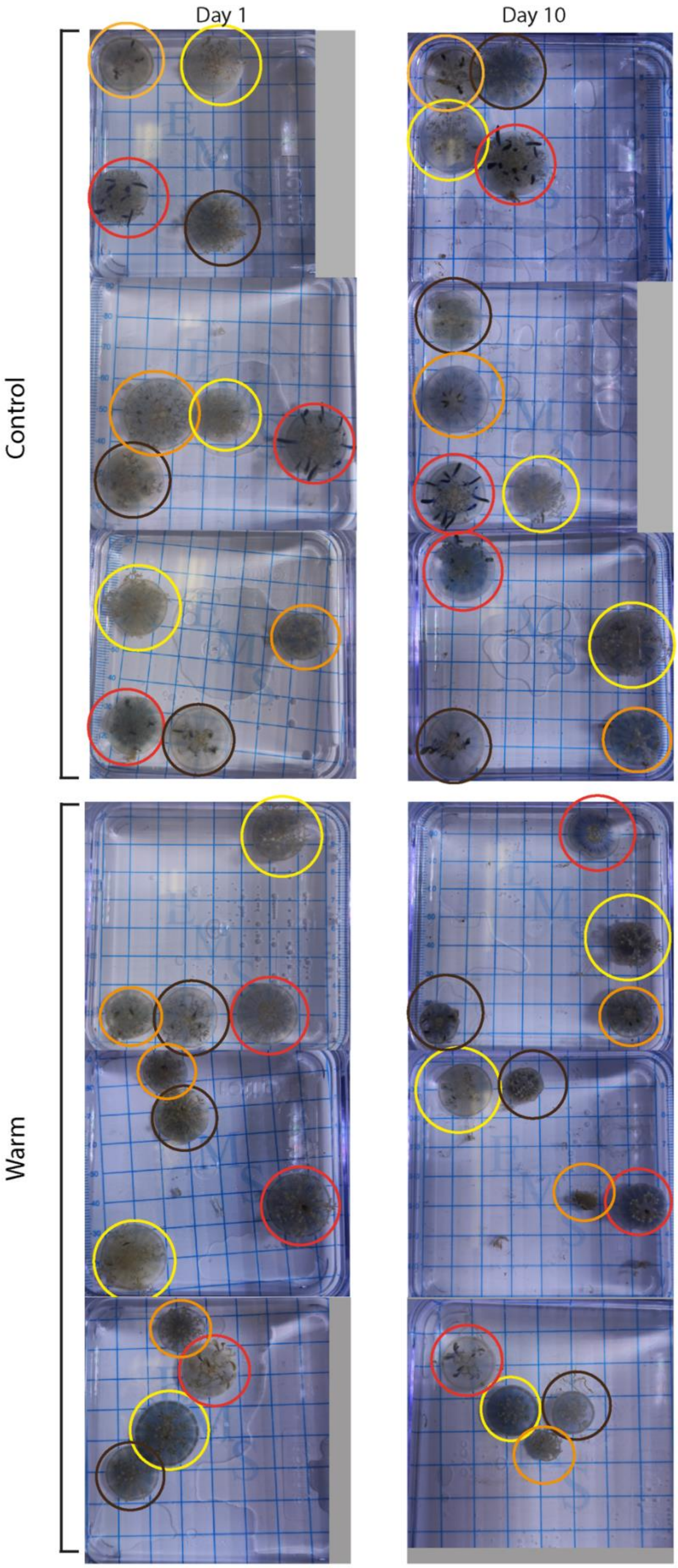

<u>Figure S2:</u> Photos of the medusae collected at SP2 on day 1 and 10 of the experiment in the control treatment, and heat stress treatment. Circles of the same color within each line indicate the same medusa.

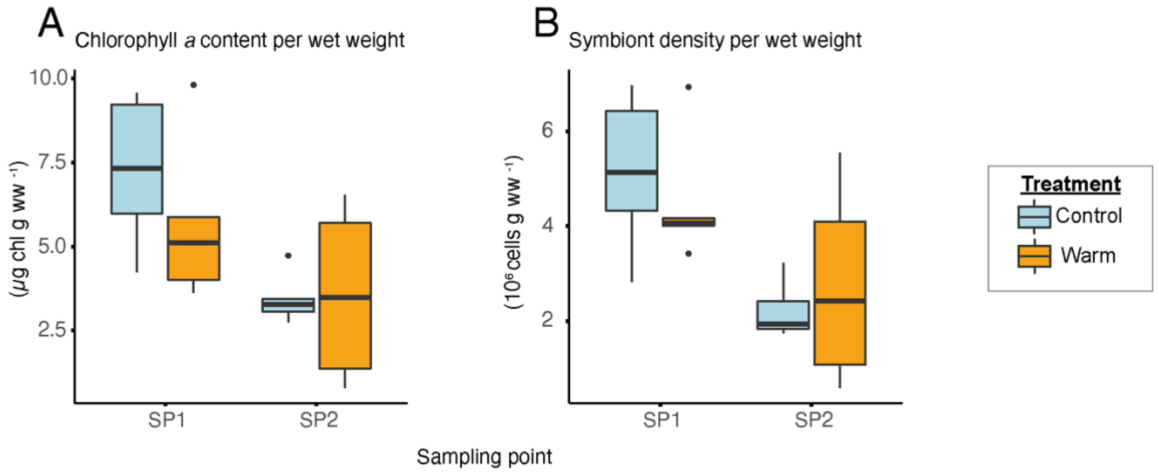

Figure S3: Impact of heat stress on chlorophyll a content (A) and algal symbiont density (B) per wet weight. Individual groups were compared using Tukey's HSD (indicated above the boxplots).

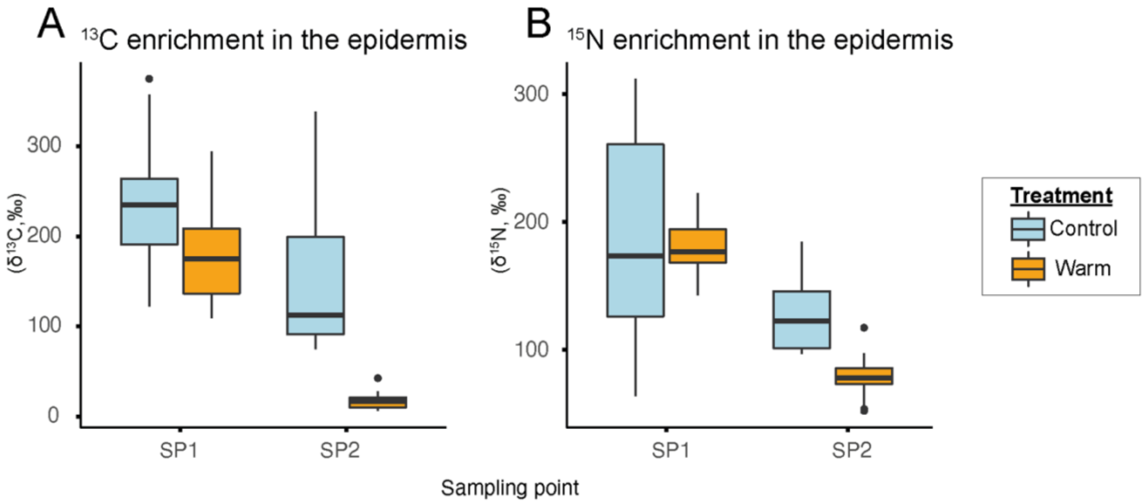

Figure S4: Temperature effects on the enrichment from assimilation of isotopically labeled bicarbonate and ammonium in the epidermis of heat-stressed medusae. ^{13}C enrichment from assimilation of ^{13}C -bicarbonate ($H^{13}C\ O_3^-$) via photosynthesis and ^{15}N induced by the assimilation of ^{15}N -ammonium ($^{15}NH_4^+$) into algal symbiont cells and host amoebocytes. Individual groups were compared using Tukey's HSD (indicated above the boxplots).

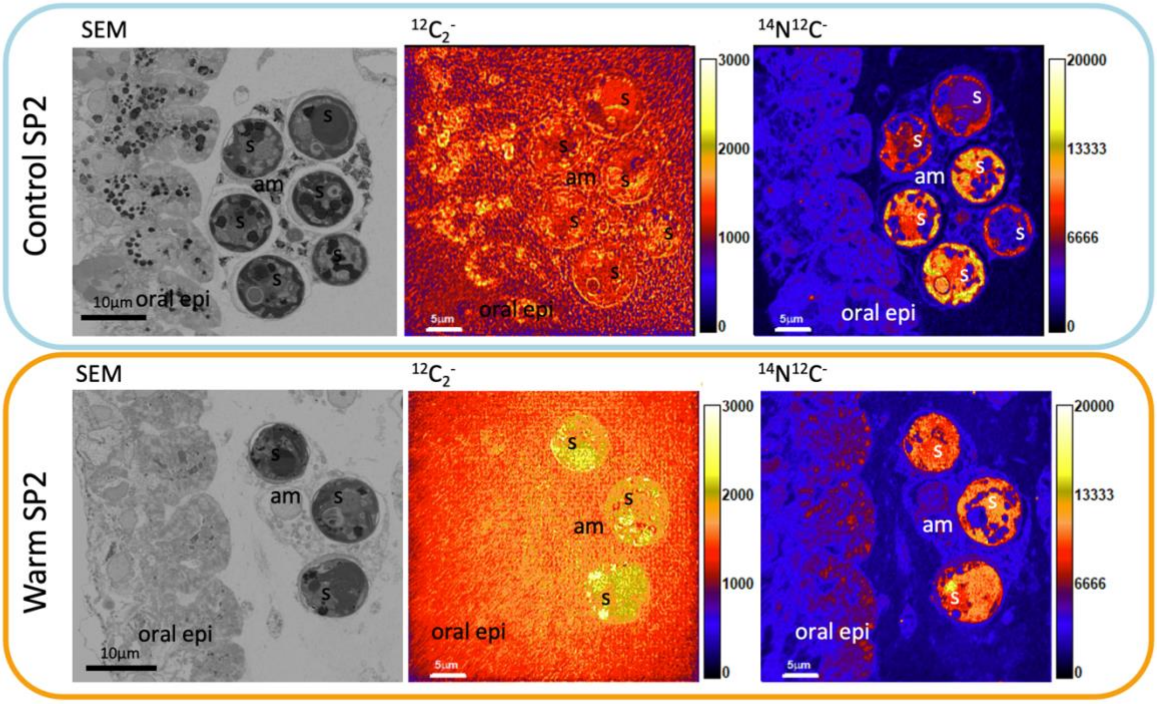

<u>Figure S5</u>: SEM and $^{12}C_2^-$ and $^{14}N^{12}C^-$ NanoSIMS images corresponding to the correlative SEM and NanoSIMS images presented in **Figure 3**. The color scales of the NanoSIMS images are linear. (s: symbiont, am: amoebocyte, oral epi: oral epidermis.)

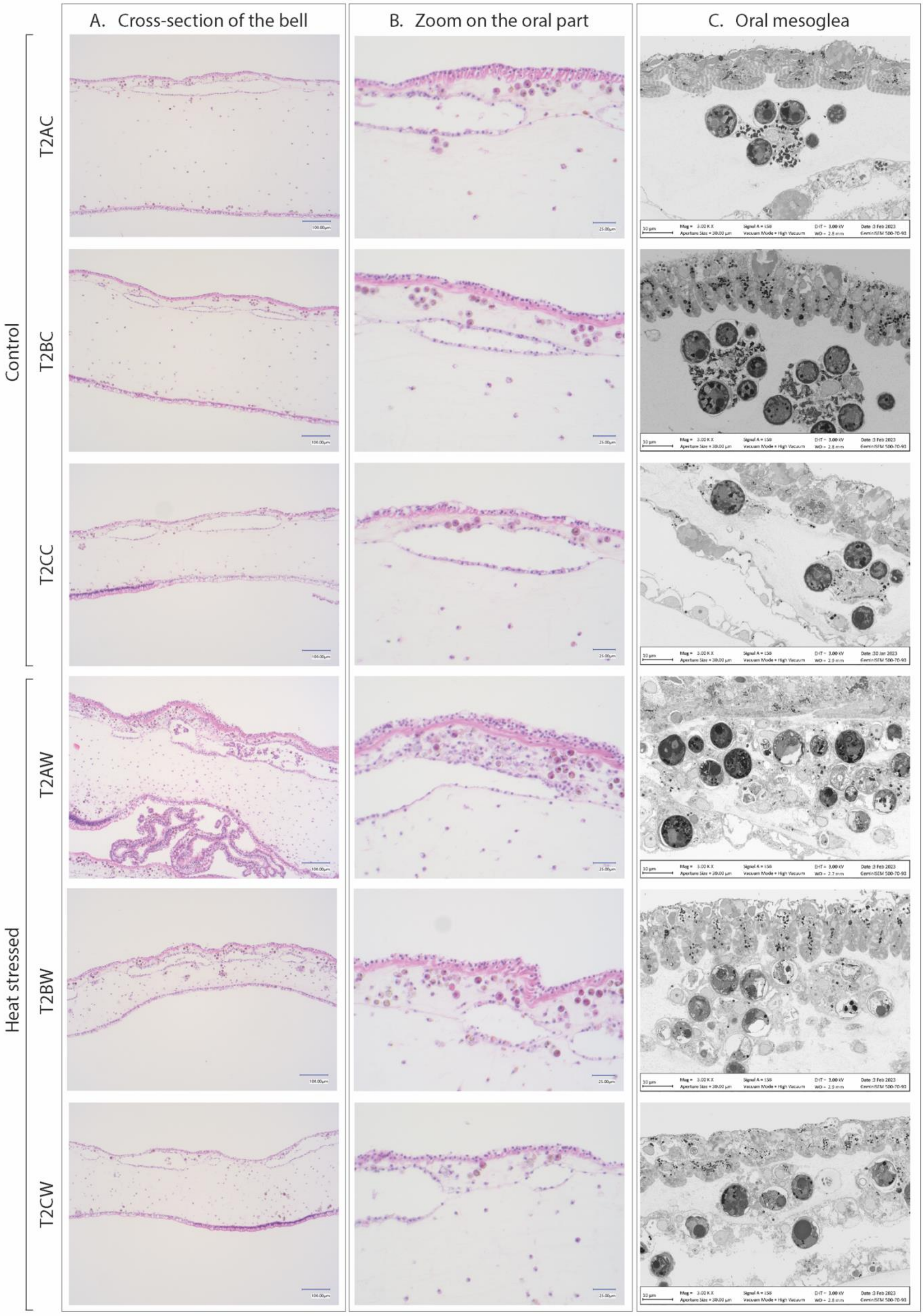
A. Cross-section of the bell
B. Zoom on the oral part
C. Oral mesoglea
Control
Heat stressed
T2AC
T2BC
T2CC
T2AW
T2BW
T2CW

<u>Figure S6:</u> Tissue and cellular ultrastructure of control and heat-stressed medusae bells at SP2 imaged by light microscopy with H&E staining (A,B) and SEM (C). Each row corresponds to one biological replicate. The annotations on the left-hand side of each row correspond to the unique sample numbers of individual replicate medusae. Scale bar **A**: 100µm, **B**: 25µm, **C**: 10µm.

<u>Table S1:</u> Number of NanoSIMS images per biological replicate and the associated number of regions of interest (ROIs) per *Cassiopea* compartment defined for the isotopic enrichment analyses.

	Biological replicates	Number of images	ROIs Symbiont	ROIs Amoebocytes	ROIs Epidermis
SP1 Control	A	7	36	8	5
	B	6	25	6	6
	C	6	29	7	5
SP1 Warm	A	5	31	6	5
	B	5	23	5	5
	C	6	25	6	5
SP2 Control	A	5	28	5	5
	B	6	33	7	4
	C	6	12	6	6
SP2 Warm	A	9	23	7	9
	B	8	34	7	5
	C	3	3	2	1
Unlabeled		26	91	24	23
